# Safety and Immunogenicity of Live Viral Vaccines in a Multicenter Cohort of Pediatric Transplant Recipients

**DOI:** 10.1001/jamanetworkopen.2023.37602

**Published:** 2023-10-12

**Authors:** Amy G. Feldman, Brenda L. Beaty, Jose A. Ferrolino, Gabriela Maron, Hillary K. Weidner, Saira A. Ali, Leandra Bitterfeld, Mary Alice Boulware, Kathleen M. Campbell, Emily Carr, Shelley Chapman, Yeh-Chung Chang, Ryan Cunningham, Ronald H. Dallas, Keerti L. Dantuluri, Bryanna N. Domenick, Noelle H. Ebel, Scott Elisofon, Rima Fawaz, Marc Foca, Hayley A. Gans, Vani V. Gopalareddy, Cindy Gu, Nitika A. Gupta, Katherine Harmann, Jessica Hollenbeck, Anna R. Huppler, Catalina Jaramillo, Nagraj Kasi, Nanda Kerkar, Stacee Lerret, Steven J. Lobritto, Maclovio J. Lopez, Elizabeth Marini, Alisha Mavis, Sonia Mehra, Lynnette Moats, Sindhu Mohandas, Flor M. Munoz, Krupa R. Mysore, Ceren Onsan, Nadia Ovchinsky, Kerrigan Perkins, Stacy Postma, Lauren Pratscher, Elizabeth B. Rand, Regina K. Rowe, Danielle Schultz, Katherine Sear, Megan L. Sell, Tanvi Sharma, Janis Stoll, Mychoua Vang, Dominique Villarin, Carly Weaver, Phoebe Wood, Olivia Woodford-Berry, George Yanni, Lara A. Danziger-Isakov

**Affiliations:** 1 Digestive Health Institute, Section of Pediatric Gastroenterology, Hepatology and Nutrition, University of Colorado and Children’s Hospital Colorado, Aurora; 2 Adult & Child Center for Outcomes Research & Delivery Science (ACCORDS), University of Colorado and Children’s Hospital Colorado, Aurora; 3 Department of Infectious Diseases, St Jude Children’s Research Hospital, Memphis, Tennessee; 4 Cincinnati Children’s Hospital Medical Center and University of Cincinnati, Cincinnati, Ohio; 5 Emory University School of Medicine, Children’s Healthcare of Atlanta, Atlanta, Georgia; 6 Intermountain Primary Children’s Hospital, Salt Lake City, Utah; 7 Levine Children’s Hospital at Atrium Health, Charlotte, North Carolina; 8 Yale University, New Haven, Connecticut; 9 Children’s Wisconsin, Medical College of Wisconsin, Milwaukee; 10 Duke University Medical Center, Durham, North Carolina; 11 NYU Grossman School of Medicine, New York, New York; 12 Children’s Hospital of Philadelphia, Philadelphia, Pennsylvania; 13 Lucile Packard Children’s Hospital at Stanford, Palo Alto, California; 14 Boston Children’s Hospital, Boston, Massachusetts; 15 Albert Einstein College of Medicine, Children’s Hospital at Montefiore, Bronx, New York; 16 Golisano Children’s Hospital at Strong, University of Rochester Medical Center, Rochester, New York; 17 C.S. Mott Children’s Hospital, Michigan Medicine, Ann Arbor; 18 Medical University of South Carolina Shawn Jenkins Children’s Hospital, Charleston; 19 Children’s Hospital of New York, NewYork-Presbyterian Hospital, New York; 20 Children’s Hospital Los Angeles, Los Angeles, California; 21 Texan Children’s Hospital, Baylor College of Medicine, Houston, Texas; 22 Washington University School of Medicine, St Louis, Missouri

## Abstract

**Importance:**

Live vaccines (measles-mumps-rubella [MMR] and varicella-zoster virus [VZV]) have not been recommended after solid organ transplant due to concern for inciting vaccine strain infection in an immunocompromised host. However, the rates of measles, mumps, and varicella are rising nationally and internationally, leaving susceptible immunocompromised children at risk for life-threating conditions.

**Objective:**

To determine the safety and immunogenicity of live vaccines in pediatric liver and kidney transplant recipients.

**Design, Setting, and Participants:**

This cohort study included select pediatric liver and kidney transplant recipients who had not completed their primary MMR and VZV vaccine series and/or who displayed nonprotective serum antibody levels at enrollment between January 1, 2002, and February 28, 2023. Eligibility for live vaccine was determined by individual US pediatric solid organ transplant center protocols.

**Exposures:**

Exposure was defined as receipt of a posttransplant live vaccine. Transplant recipients received 1 to 3 doses of MMR vaccine and/or 1 to 3 doses of VZV vaccine.

**Main Outcome and Measure:**

Safety data were collected following each vaccination, and antibody levels were obtained at 0 to 3 months and 1 year following vaccination. Comparisons were performed using Mann-Whitney *U* test, and factors associated with development of postvaccination protective antibodies were explored using univariate analysis.

**Results:**

The cohort included 281 children (270 [96%] liver, 9 [3%] kidney, 2 [1%] liver-kidney recipients) from 18 centers. The median time from transplant to enrollment was 6.3 years (IQR, 3.4-11.1 years). The median age at first posttransplant vaccine was 8.9 years (IQR, 4.7-13.8 years). A total of 202 of 275 (73%) children were receiving low-level monotherapy immunosuppression at the time of vaccination. The majority of children developed protective antibodies following vaccination (107 of 149 [72%] varicella, 130 of 152 [86%] measles, 100 of 120 [83%] mumps, and 124 of 125 [99%] rubella). One year post vaccination, the majority of children who initially mounted protective antibodies maintained this protection (34 of 44 [77%] varicella, 45 of 49 [92%] measles, 35 of 42 [83%] mumps, 51 of 54 [94%] rubella). Five children developed clinical varicella, all of which resolved within 1 week. There were no cases of measles or rubella and no episodes of graft rejection within 1 month of vaccination. There was no association between antibody response and immunosuppression level at the time of vaccination.

**Conclusions and Relevance:**

The findings suggest that live vaccinations may be safe and immunogenic after solid organ transplant in select pediatric recipients and can offer protection against circulating measles, mumps, and varicella.

## Key Points

**Question:**

What are the safety and immunogenicity of live vaccines (measles-mumps-rubella and varicella-zoster virus) in select pediatric solid organ transplant recipients?

**Findings:**

In this cohort study of 281 pediatric liver and kidney transplant recipients from 18 US transplant centers, no serious adverse events were observed following live vaccination, with the majority of children developing protective antibodies (72% varicella, 86% measles, 83% mumps, and 99% rubella).

**Meaning:**

These findings suggest that administration of live vaccines to select transplant recipients can offer seroprotection against the ongoing risk of exposure to circulating measles, mumps, and varicella.

## Teaser

This cohort study assesses the safety and immunogenicity of live viral vaccines in pediatric liver and kidney transplant recipients.

## Introduction

Historically, live vaccines have not been recommended after solid organ transplant (SOT) due to a theoretical risk of vaccine strain infection in an immunocompromised host.^1^ Global surges of measles and mumps, exacerbated by decreasing herd immunity as millions of vaccine doses were missed during the SARS-CoV-19 pandemic, leave nonimmune SOT recipients at significant risk for community exposure, infection, morbidity, and mortality from wild-type infection.^2,3,4^ Previous small, single-center studies dating back to the early 1990s have demonstrated the safety and efficacy of live viral vaccines in highly select pediatric SOT recipients under controlled settings.^5,6,7,8,9,10^ On the basis of these studies, in 2019, the American Society of Transplantation and the International Pediatric Transplant Association published novel recommendations that live vaccines (measles-mumps-rubella [MMR] vaccine and varicella-zoster virus [VZV] vaccine) should be considered for select nonimmune SOT recipients more than 1 year post transplant who are receiving low-level immunosuppression.^11,12^ However, a 2020 survey study assessing posttransplant live vaccine practices at individual Society of Pediatric Liver Transplantation centers found that despite these recommendations, only 29% (12 of 41) of centers were routinely offering live vaccines post transplant.^13^ Following this survey study, 18 pediatric SOT centers from the Society of Pediatric Liver Transplantation and the Pediatric Infectious Diseases Society joined together to administer live vaccines to nonimmune SOT recipients as part of the LIVE VAC cohort study. This study assesses the safety and immunogenicity of live vaccines in pediatric SOT recipients participating in the LIVE VAC study.

## Methods

This cohort study was reviewed by each participating center’s institutional review board. Oral and/or written consent was obtained from participants when required by the individual center (ie, some centers used deidentified data). The study followed the Strengthening the Reporting of Observational Studies in Epidemiology (STROBE) reporting guideline for cohort studies.^14^

### LIVE VAC Study Design and Population

The LIVE VAC study cohort includes pediatric liver and kidney transplant recipients from 18 participating institutions across the US who received posttransplant live vaccines (MMR and/or VZV) between January 1, 2002, and February 28, 2023. Enrolled patients either had not completed their primary MMR and VZV vaccine series and/or displayed nonprotective serum antibody levels at the time of enrollment. Eligibility for posttransplant live vaccines was determined by individual center protocols (eTable in Supplement 1). Demographic, clinical, and laboratory data were collected pre- and postvaccination according to individual center clinical practice guidelines. Data on sex, race, and ethnicity were not collected as they were not relevant to the study question.

### Study Outcomes

The primary outcome was SOT recipient development of seroprotective antibodies against MMR and VZV following posttransplant live vaccination. Seroprotective antibody levels were determined by each center’s individual laboratory methods and values. Secondary outcomes included serious adverse outcome following vaccine administration, development of clinical disease in the year following vaccine administration, and persistence of seroprotective antibodies at 1-year postvaccine administration.

### Statistical Analysis

Continuous variables are presented as medians and IQRs. Comparison of demographic characteristics between groups was performed using the Mann-Whitney *U* test. Categorical variables are presented as absolute numbers and percentages, and the χ^2^ or Fisher exact test, as appropriate, was used to compare the groups. Univariate analysis was performed to explore factors associated with development of postvaccination protective antibodies. The statistical analysis was conducted using SAS, version 9.4 software (SAS Institute Inc). Statistical significance was set at *P* < .05 by 2-sided tests.

## Results

A total of 281 children (270 [96%] liver, 9 [3%] kidney, and 2 [1%] liver and kidney recipients) from 18 centers received 1 or more doses of live vaccine after SOT (45 [16%] VZV only, 64 [23%] MMR only, and 172 [61%] both for a total of 236 children receiving MMR and 217 receiving VZV vaccines). The median time from transplant to enrollment was 6.3 years (IQR, 3.4-11.1 years). The median age at first posttransplant study vaccine was 8.9 years (IQR, 4.7-13.8 years) (Table 1).

**Table 1.** Characteristics of All Participants (n = 281)

Characteristic	No. (%)
Posttransplant vaccine received	
MMR only	64 (23)
VZV only	45 (16)
MMR and VZV	172 (61)
Organ type	
Liver	270 (96)
Kidney	9 (3)
Liver and kidney	2 (1)
Age at transplant, median (IQR), y	0.9 (0.6-1.7)
Age at first posttransplant vaccine, median (IQR), y	8.9 (4.7-13.8)
Time between transplant and enrollment, median (IQR), y	6.3 (3.4-11.1)
History of at least 1 dose of pretransplant vaccine	
MMR	102 (36)
VZV	95 (34)
History of preenrollment use of thymoglobulin, rituximab, or alemtuzumab biologic	15 (5)
History of receiving blood products within the year of enrollment	11 (4)
History of any rejection in the 2 y before enrollment	35 (12)
Epstein-Barr viral DNA quantification enrollment (n = 218)	
Negative or <2000 IU/mL	203 (93)
≥2000 IU/mL	15 (7)
Age-appropriate absolute lymphocyte count at enrollment, lymphocytes/μL (n = 244)	240 (98)
Age-appropriate immunoglobulin G level, mg/dL (n = 127)	124 (98)
Age-appropriate CD4 count, cells/μL (n = 82)	71 (87)
Immunosuppression at enrollment (n = 275)	
Low: monotherapy with tacrolimus (trough <5 ng/mL), sirolimus (trough <5 ng/mL), or cyclosporine (trough <100 ng/mL)	202 (73)
Medium: ≤2 agents and/or tacrolimus plus sirolimus trough between 5 and 8 ng/mL, and/or steroids <0.5 mg/kg per dose	39 (14)
High^a^: ≥3 agents and/or tacrolimus plus sirolimus trough >8 ng/mL, and/or steroids ≥0.5 mg/kg/d	34 (12)

Abbreviations: MMR, measles-mumps-rubella; VZV, varicella-zoster virus.

^a^ Immunosuppressive agents used in addition to tacrolimus, sirolimus, cyclosporine, and steroids include azathioprine (5 participants [2%]) and mycophenolate mofetil (19 participants [7%]).

### Pretransplant Live Vaccination History

Pretransplant, 95 of 281 (34%) children received the VZV vaccine, and 102 of 281 (36%) received the MMR vaccine (Table 1). Of those children who received pretransplant live vaccines and subsequently had pretransplant antibodies rechecked, there was variable development of pretransplant protection (VZV, 22 of 50 [44%]; measles, 24 of 34 [71%]; mumps, 16 of 27 [59%]; rubella, 21 of 30 [70%]). This protection was not consistently maintained post transplant. At study enrollment, of those children who had documented protective pretransplant antibodies and then had antibodies rechecked, 5 of 20 (25%) maintained protection for VZV, 11 of 15 (73%) for measles, 10 of 14 (71%) for mumps, and 15 of 18 (83%) for rubella (Table 2).

**Table 2.** Immunologic Response to Pretransplant and Posttransplant Live Viral Vaccines

Time of immunologic assessment	No./total No. (%) with protective titers			
	Varicella	Measles	Mumps	Rubella
After pretransplant vaccine^a^	22/50 (44)	24/34 (71)	16/27 (59)	21/30 (70)
At enrollment after protective pretransplant antibodies^b^	5/20 (25)	11/15 (73)	10/14 (71)	15/18 (83)
After first posttransplant vaccine^c^	53/116 (46)	92/129 (71)	62/101 (61)	100/106 (94)
After second posttransplant vaccine^c^	61/81 (75)	48/60 (80)	41/52 (79)	43/44 (98)
After third posttransplant vaccine^c^	5/9 (56)	3/6 (50)	5/6 (83)	5/5 (100)
After final posttransplant vaccine^c^	107/149 (72)	130/152 (86)	100/120 (83)	124/125 (99)
1 y Postvaccine^d^	34/44 (77)	45/49 (92)	35/42 (83)	51/54 (94)

^a^ Participants who received pretransplant vaccine and subsequently had pretransplant antibodies checked.

^b^ Participants who had protective pretransplant antibodies and subsequently had antibodies checked at enrollment (before posttransplant vaccine).

^c^ Participants who received a posttransplant vaccine and subsequently had posttransplant antibodies checked.

^d^ Participants who had protective antibodies after receiving posttransplant vaccine and then subsequently had antibodies checked at 1 y after initial vaccine.

### Posttransplant Live Vaccination

At enrollment, 205 of 217 (94%) children who later received a VZV vaccination as part of the study were VZV nonimmune (either no history of VZV immunization or documented negative VZV antibodies at enrollment). At enrollment, 229 of 236 (97%) children who later received an MMR vaccination as part of the study either had no history of MMR immunization or were negative for measles, mumps, and/or rubella antibodies. The majority of children were receiving low-level monotherapy immunosuppression at enrollment (202 of 275 [73%]) (Table 1). However, 34 of 275 (12%) children were receiving high-level immunosuppression defined as a tacrolimus or sirolimus trough greater than 8 ng/mL (n = 13), and/or steroids greater than or equal to 0.5 mg/kg/d (n = 2), and/or mycophenolate mofetil (n = 19), and/or azathioprine (n = 5). Of the 281 children who had received posttransplant MMR, VZV, or both vaccines and subsequently had antibodies tested (most frequently 1-3 months post vaccination), protective antibodies were present at varying levels (VZV, 107 of 149 [72%]; measles, 130 of 152 [86%]; mumps, 100 of 120 [83%]; rubella, 124 of 125 [99%]). At 1 year post vaccination, the majority of children who initially mounted protective antibodies maintained protection (VZV, 34 of 44 [77%]; measles, 45 of 49 [92%]; mumps, 35 of 42 [83%]; rubella, 51 of 54 [94%]) (Table 2). Following VZV vaccination, 5 of 217 (2%) children developed clinical varicella (all ≥7 days postvaccine), with all conditions resolving within 1 week (3 with antiviral therapy). All 5 children were receiving medium or high immunosuppression. Transient nontender subauricular lymph node swelling occurred 3 weeks after MMR vaccination in 1 patient, raising concern for mumps; however, the condition resolved without intervention. There were no measles or rubella cases and no organ rejection episodes within 1 month of vaccination. An association was found between development of protective measles antibodies and level of immunosuppression (medium or low immunosuppression, 17 of 22 [77%] and 118 of 128 [92%] below and equal to or above the level of protection, respectively; *P* = .047), but no associations were found between development of protective antibodies and age at transplant, pretransplant vaccination status, Epstein-Barr virus level at enrollment, or absolute lymphocyte count at enrollment (Table 3).

**Table 3.** Characteristics of Participants by Antibody Response to Posttransplant Live Vaccines

Variable^a^	No./total No. (%)		*P* value^b^
	Below the level of protection	Equal to or above the level of protection	
**Varicella antibody response (n = 149)**			
Age at transplant, median (IQR), y	0.8 (0.5-1.3)	1.0 (0.6-1.9)	.36^c^
Time between transplant and study enrollment, median (IQR), y	4.7 (2.4-11.1)	6.6 (4.1-10.6)	.09^c^
Patient received VZV vaccine before transplant	15/42 (36)	40/107 (37)	.85
Epstein-Barr virus PCR (n = 131)			
Negative or <2000 IU/mL	36/37 (97)	83/94 (88)	.18^d^
≥2000 IU/mL	1/37 (3)	11/94 (12)	
Age-appropriate level of ALC (n = 137)	38/39 (97)	98/98 (100)	.28^d^
Immunosuppression level (n = 146)^e^			
High	7/42 (17)	15/104 (14)	.73
Medium or low	35/42 (83)	89/104 (86)	
No. of VZV vaccines received			
1	19/42 (45)	33/107 (31)	.02
2	16/42 (38)	67/107 (63)	
3	7/42 (17)	7/107 (7)	
**Measles antibody response (n = 152)**			
Age at transplant, median (IQR), y	1.0 (0.8-1.3)	1.0 (0.6-1.6)	.58^c^
Time between transplant and study enrollment, median (IQR), y	5.6 (3.5-10.0)	7.6 (4.7-11.2)	.28^c^
Patient received MMR vaccine before transplant	11/22 (50)	43/130 (33)	.13
Epstein-Barr virus PCR (n = 123)			
Negative or<2000 IU/mL	16/16 (100)	98/107 (92)	.60^d^
≥2000 IU/mL	0/16	9/107 (8)	
Age-appropriate level of ALC (n = 145)	20/21 (95)	122/124 (98)	.38^d^
Immunosuppression level (n = 150)^e^			
High	5/22 (23)	10/128 (8)	.047^d^
Medium or low	17/22 (77)	118/128 (92)	
No. of MMR vaccines received			
1	8/22 (36)	65/130 (50)	.12
2	10/22 (45)	57/130 (44)	
3	4/22 (18)	8/130 (6)	
**Mumps antibody response (n = 120)**			
Age at transplant, median (IQR), y	1.0 (0.7-1.5)	1.0 (0.6-1.6)	.76^c^
Time between transplant and study enrollment, median (IQR), y	9.6 (4.2-14.7)	6.7 (4.3-11.1)	.38^c^
Patient received MMR vaccine before transplant	5/20 (25)	34/100 (34)	.43
Epstein-Barr virus PCR (n = 107)			
Negative or <2000 IU/mL	15/17 (88)	84/90 (93)	.61^d^
≥2000 IU/mL	2/17 (12)	6/90 (7)	
Age-appropriate level of ALC (n = 111)	19/19 (100)	91/92 (99)	.99^d^
Immunosuppression level (n = 118)^e^			
High	3/19 (16)	13/99 (13)	.72^d^
Medium or low	16/19 (84)	86/99 (87)	
No. of MMR vaccines received			
1	7/20 (35)	48/100 (48)	.56
2	11/20 (55)	43/100 (43)	
3	2/20 (10)	9/100 (9)	

Abbreviations: ALC, absolute lymphocyte count; MMR, mumps-measles-rubella; PCR, polymerase chain reaction; VZV, varicella-zoster virus.

^a^ Rubella comparisons not included as all but 1 participant had a positive response to vaccination.

^b^ χ^2^ Test unless otherwise noted.

^c^ Mann-Whitney *U* test.

^d^ Fisher exact test.

^e^ Low immunosuppression: monotherapy with tacrolimus (trough <5 ng/mL), sirolimus (trough <5 ng/mL), or cyclosporine (trough <100 ng/mL). Medium immunosuppression: 2 or fewer agents or tacrolimus plus sirolimus trough between 5 and 8 ng/mL or steroids <0.5 mg/kg per dose. High immunosuppression: 3 or more agents or tacrolimus plus sirolimus trough >8 ng/mL or steroids ≥0.5 mg/kg/d.

## Discussion

In this cohort study of 281 pediatric liver and kidney transplant recipients across the US, live viral vaccination was found to be safe and immunogenic. There were no serious adverse events that resulted in graft injury or patient death. The majority of children developed protective antibodies after receiving between 1 and 3 doses of MMR, VZV, or both vaccines. While the majority of children in the study met recommended criteria by Suresh et al^11^ and the American Society of Transplantation^12^ for post-SOT live vaccines (low level immunosuppression, >1 year post transplant, >2 months postrejection, age-appropriate absolute lymphocyte count), there were children in this study outside of these criteria. Additionally, there were children in this cohort who were receiving mycophenolate mofetil and who had positive Epstein-Barr virus levels at the time of vaccination, criteria that met Suresh et al’s recommendation to vaccinate with caution.^11^ In univariate analysis, overall immunosuppression level, time since transplant, Epstein-Barr virus levels, and mycophenolate mofetil use were not associated with development of protective antibodies. These data suggest that eligibility criteria for posttransplant live vaccination may not need to be as stringent, and a larger number of nonimmune pediatric SOT recipients could potentially be candidates for posttransplant live vaccination.

### Limitations

This study was limited by its observational nature. Each center determined criteria for vaccine eligibility and timing of postvaccine laboratory testing. However, this variability allowed for evaluation of a heterogeneous population of transplant recipients receiving different levels of immunosuppression and at different intervals post transplant. A second limitation was incomplete data on pretransplant vaccine history and pretransplant antibody levels, making it difficult to fully understand the association between pretransplant live vaccination and posttransplant response to live vaccination. There is wide center variation in both pretransplant administration of live vaccines and pretransplant evaluation of MMR and VZV antibodies.^15^ A third limitation was the small number of kidney transplant recipients included in the study. Finally, not all children in the study had 1-year titers drawn; therefore, conclusions about maintenance of protective antibodies after vaccination are incomplete. Likewise, we can only report development of disease up to 1 year. In future studies, we hope to expand the enrolled population (including more kidney transplant recipients) and the follow-up period.

## Conclusions

The findings of this cohort study suggest that further research is needed to understand long-term maintenance of immunity after live post-SOT vaccination in pediatric recipients, as well as factors associated with immune response and clinical protection. These data may help define appropriate candidates, ideal timing, and dosing intervals for live vaccination after SOT in pediatric populations. Dissemination of safety and immunogenicity data and sharing of effective implementation strategies and outcomes could be important to accelerate adoption of these novel recommendations across SOT centers.

We are no longer living in the measles-free US of the early 2000s. Measles outbreaks are under way, and nonimmune, immunocompromised children could be at risk of virus acquisition. Live vaccine administration post-SOT is a feasible intervention to prevent morbidity and mortality in pediatric SOT recipients and should be strongly encouraged in appropriate patients.

## Notes

Supplement 1. **eTable.** Individual Transplant Center Criteria for Posttransplant Live Vaccine Eligibility (N = 18)

Supplement 2.
Data Sharing Statement
