# Supplementary material for: Safety and Immunogenicity of Live Viral Vaccines in a Multicenter Cohort of Pediatric Transplant Recipients
Source: JAMA Netw Open. 2023 Oct 12;6(10):e2337602. doi: 10.1001/jamanetworkopen.2023.37602 (PMC10570873; doi:10.1001/jamanetworkopen.2023.37602)
Supplement: Supplement 1. — eTable. Individual Transplant Center Criteria for Posttransplant Live Vaccine Eligibility (N = 18) [file jamanetwopen-e2337602-s001.pdf]

Supplemental Online Content

Feldman AG, Beaty BL, Ferrolino JA, et al. Safety and immunogenicity of live viral vaccines in a multicenter cohort of pediatric transplant recipients. *JAMA Netw Open*. 2023;6(10):e2337602. doi:10.1001/jamanetworkopen.2023.37602

eTable. Individual Transplant Center Criteria for Posttransplant Live Vaccine Eligibility (N = 18)

This supplemental material has been provided by the authors to give readers additional information about their work.

© 2023 Feldman AG et al. *JAMA Network Open.*

eTable. Individual Transplant Center Criteria for Posttransplant Live Vaccine Eligibility (N = 18)

Eligibility Criteria	No (%)
Live vaccine history and/or antibodies at enrollment	
Negative antibodies required at enrollment	10 (56)
No history of any live vaccines or negative antibodies required at enrollment	2 (11)
Incomplete history of live vaccines or negative antibodies required at enrollment	6 (33)
Single organ transplant only	14 (78)
Time from transplant (months)	
6-11 months	1 (6)
12-17 months	12 (67)
18-23 months	3 (17)
24+ months	1 (6)
Not specified	1 (6)
Time from acute cellular rejection (months)	
2- 5 months	4 (22)
6-11 months	8 (44)
12+ months	2 (11)
Not specified	4 (22)
Epstein Barr Virus PCR	
Undetectable	2 (11)
<5000 IU/mL	5 (28)
No specific level required or not routinely assessed	11 (61)
Cytomegalovirus PCR	
Undetectable	4 (22)
<5000 IU/mL	2 (11)
No specific level required or not routinely assessed	12 (67)
Time from thymoglobulin (ATG)	
No history of ATG	4 (22)
At least 6 months	2 (11)
At least 12 months	7 (39)
Not specified	5 (28)
Time from rituximab	
No history of rituximab	4 (22)
At least 6 months	2 (11)
At least 12 months	8 (44)
Not specified	4 (22)
Time from Alemtuzumab	
No history of Alemtuzumab	5 (28)
At least 6 months	1 (6)
At least 24 months	6 (33)
Not specified	6 (33)
Time from intravenous immunoglobulin (IVIG)	
Per Red Book/Centers for Disease Control and Prevention	13 (72)
Not specified	5 (28)
Time from blood transfusion	
At least 6 months	12 (67)
Not specified	6 (33)
Absolute lymphocyte count	
>750 cells/μL	1 (6)
Normal for age	10 (56)
No specific requirement	7 (39)
CD4 level	
500-700 cells/mm^3	6 (33)

© 2023 Feldman AG et al. *JAMA Network Open.*

No specific requirement	12 (67)
Immunoglobulin G level	
75% of normal for age	1 (6)
Normal for age	7 (39)
No specific requirement	10 (56)
Single immunosuppressive agent	4 (22)
Maximum tacrolimus, sirolimus, or combined trough level	
0-5.9	8 (44)
6-7.9	8 (44)
No specific	2 (11)
Steroids permitted	
Yes	11 (61)
Imuran permitted	
Yes	5 (28)
Cellcept permitted	
Yes	6 (33)

Individual centers also specified no serious infections resulting in hospitalization in the past 6 months (2 centers), reliable history of phone communication (2 centers), no chemotherapy in the last 12 months (1 center), no patients with history of re-transplantation (1 center), no COVID in the past 6 months (1 center), no post-transplant lymphoproliferative disease in the last 6 months (1 center)

© 2023 Feldman AG et al. *JAMA Network Open.*
