# Supplementary material for: Safety and Immunogenicity of Live Viral Vaccines in a Multicenter Cohort of Pediatric Transplant Recipients
Source: JAMA Netw Open. 2023 Oct 12;6(10):e2337602. doi: 10.1001/jamanetworkopen.2023.37602 (PMC10570873; doi:10.1001/jamanetworkopen.2023.37602)
Supplement: Supplement 2. — Data Sharing Statement [file jamanetwopen-e2337602-s002.pdf]

Data Sharing Statement

Feldman. Safety and Immunogenicity of Live Viral Vaccines in a Multicenter Cohort of Pediatric Transplant Recipients. *JAMA Netw Open*. Published October 12, 2023.
doi:10.1001/jamanetworkopen.2023.37602

Data
Data available: Yes
Data types: Data (not involving human participants), Data dictionary, Deidentified participant data
How to access data: available from author
When available: With publication

Supporting Documents
Document types: Statistical/analytic code
How to access documents: available from author
When available: With publication

Additional Information
Who can access the data: none
Types of analyses: any purpose
Mechanisms of data availability: from author with a signed data access agreement
Any additional restrictions: none
